# Response to Bifulco Comments on Triassi *et al.* Environmental Pollution from Illegal Waste Disposal and Health Effects: A Reviewer on the “Triangle of Death”. *Int. J. Environ. Res. Public Health* 2015, *12*, 1216–1236

**DOI:** 10.3390/ijerph120303360

**Published:** 2015-03-19

**Authors:** Maria Triassi, Rossella Alfano, Maddalena Illario, Antonio Nardone, Oreste Caporale, Paolo Montuori

**Affiliations:** 1 Department of Public Health, “Federico II” University, Naples 80131, Italy; E-Mails: triassi@unina.it (M.T.); rossellaalfan@gmail.com (R.A.); antonio.nardone@unina.it (A.N.); oreste.caporale@libero.it (O.C.); 2 Department of Traslational Medical Science, “Federico II” University, Naples 80131, Italy; E-Mail: illario@unina.it

We thank Bifulco [1] for his interest in reading our article and his time in writing his comments on our work [2]. Our response to his concerns are as follows:

The diseases studies in the papers that we cited in our Review on the Triangle of Death, and also others we did not include because they did not meet the inclusion criteria (Scopus, *etc.*), have a long period of latency. Environmental exposure related diseases require a long time before a diagnosis occurs. Diagnosis is made when the diseases ascertained, but latency refers to the length of time it takes from being exposed until when the disease becomes apparent in a clinical examination.

Latency can be as short as 10 years or as long as 50, but the average length of latency for the diseases we studied is about 20 years between exposure and diagnosis (average for malignant mesothelioma is 35 to 40 years between exposure and diagnosis).

The application of genetics to disease susceptibility moves away from the clinic towards the population, that integrated with the observational methods of epidemiology can potentially support more effective public health measures.

The majority of genetic variants or single nucleotide polymorphisms in the human genome are of low penetrance, also for genes involved in metabolism of environmental chemicals, immunity, lipid metabolism, and hemostasis among others. The high prevalence of these single nucleotide polymorphisms means that, despite the low penetrance, they could provide a significant contribution to population disease burden. Exposure-disease associations and the interplay with genetic susceptibility requires further fundamental knowledge and studies on genetic variation, environment, lifestyle, and chronic disease, that are pivotal to provide insights into disease etiology at the population level. In alignment with this vision, Prof. Bifulco reports: “In my opinion, despite preliminary scientific studies suggest a link between hazardous exposure from toxic wastes and cancer occurrence [4], to definitively clarify this point further extensive and analytical studies are required”. Indeed, Barba *et al.* [3] conclude that currently available evidence suffers from limitations mainly due to study design, lack of consideration of confounders and quality of the exposure data.

Despite all the sufferings and the concerns that the many issues and implications linked to the Land of Fires raise, it still provides an invaluable opportunity for research. The characterization of pollution types and sources would support effective and targeted measures to eliminate and reduce associated health risks, contributing to improved health outcomes for the population.
